# Efficacy of Ropivacaine for Sub-Arachnoid Block in Patients with Recent History of Scorpion Sting: A case series

**DOI:** 10.18295/squmj.3.2024.022

**Published:** 2024-05-27

**Authors:** Saurabh Trivedi, Hemendra Bhardwaj, Tapan K. Sahoo, Seema Gupta

**Affiliations:** Department of Anaesthesia & Critical Care, Chirayu Medical College & Hospital, Bhopal, India

**Keywords:** Bupivacaine, Ropivacaine, Scorpion Stings, Spinal Anaesthesia

## Abstract

Failure of sub-arachnoid block (SAB), due to resistance to bupivacaine after a recent scorpion sting can lead to multiple block attempts and subsequent conversion to general anaesthesia. We report this case series of 10 patients with successful SAB with newly launched 0.75% hyperbaric ropivacaine, in patients with recent scorpion sting. Thus, intrathecal hyperbaric ropivacaine may be considered as the local anaesthetic agent of choice in patients with scorpion sting to prevent failure of SAB.

Spinal anaesthesia or sub-arachnoid block (SAB) is a commonly practised technique of anaesthesia for most below-umbilical procedures.^1^ Clinical data has shown a correlation between previous history of a scorpion sting and resistance to SAB with bupivacaine, an amino-amide local anaesthetic.^2^^,^^3^

Ropivacaine(2,6-dimethylphenyl, 1-propylpiperidine, 2-carboxamide) is a piperidine-carboxamide-based amide, prepared as pure S-enantiomer.^4^ Multiple studies have shown that ropivacaine is a safe and effective local anaesthetic (LA) for regional anaesthesia techniques. Hyperbaric preparation of ropivacaine (0.75%) for SAB have recently been launched in India, and its efficacy and safety for intra-thecal administration has been documented.^5^

We report successful SAB with ropivacaine in patients with confirmed history of a recent scorpion sting.

## Case Series

This case series includes 10 patients of either gender, aged 18–70 years, with a history of a scorpion sting within 5 years, undergoing below umbilical surgeries under SAB, between August 2022 and November 2022 at a tertiary care hospital of central India [Table 1]. Patients with confirmed history of scorpion sting in pre-anaesthetic check-up, were further evaluated for the number of stings, duration since last sting and severity of the sting—grade 1: local pain and paraesthesia at the sting site; grade 2: local pain and paraesthesia existing at the sting site as well as proximal to the sting site; grade 3: grade 2 factors with added cranial nerve (increased oral secretions, blurry vision, rapid tongue movement, nystagmus) or skeletal neuromuscular dysfunction (flailing of the extremities and tetanus-like arching of the back) with or without autonomic dysfunction; grade 4: includes both cranial nerve and skeletal muscle dysfunction, hyperthermia, rhabdomyolysis, pulmonary oedema, multiple organ failures.^6^

After standard fasting of 8 hours, patients were shifted to the operation theatre and standard monitors were applied and baseline vitals were noted. A single operator performed all the SAB, using 25 gauge, 90 mm Becton Dickinson (BD), Quinke needle, at L3–4 interspace in the sitting position, with a standard dose of 3.2 mL, 0.75% hyperbaric ropivacaine. The patients were positioned supine immediately after drug administration and assessment for autonomic, sensory and motor blockage was done by a blinded observer (trained anaesthesiologist) immediately after supine positioning.

Haemodynamic parameters were noted every minute for 5 minutes from the time of supine positioning and every 5 minutes afterwards for 20 minutes.

Sensory blockage was assessed using pinprick method, using a two-point scoring system (0 = normal sensation; 1 = loss of pain sensation but pressure sensation intact; 2 = loss of pain and pressure sensation). A score of 1 and 2 at T10 level, were considered as onset and completion of sensory block, respectively.

Bromage scale was used for motor blockage assessment.^7^ Bromage grade II and IV were considered as onset and completion of motor block, respectively. The block was considered adequate when a complete sensory and motor blockage was achieved at T10 level and planned surgery was started. In case of inadequate blockage (a sensory score of 0 or 1 with Bromage grade <IV) at 20 minutes after SAB, it was considered a block failure. On the completion of surgery, patients were shifted to post-anaesthesia care units for monitoring.

The time for onset and completion of sensory and motor blockage and block failure were assessed for association with number, duration and severity of sting.

A written-informed consent was obtained from all the patients for publication purposes.

## Results

The mean time to onset of sensory and motor block was 78.8 and 94.2 seconds, respectively. The mean time to completion of sensory and motor blockage was 117 and 146.7 seconds, respectively. All 10 patients achieved complete sensory and motor blockage within 20 minutes and none of the SAB failed.

**Figure 1:**
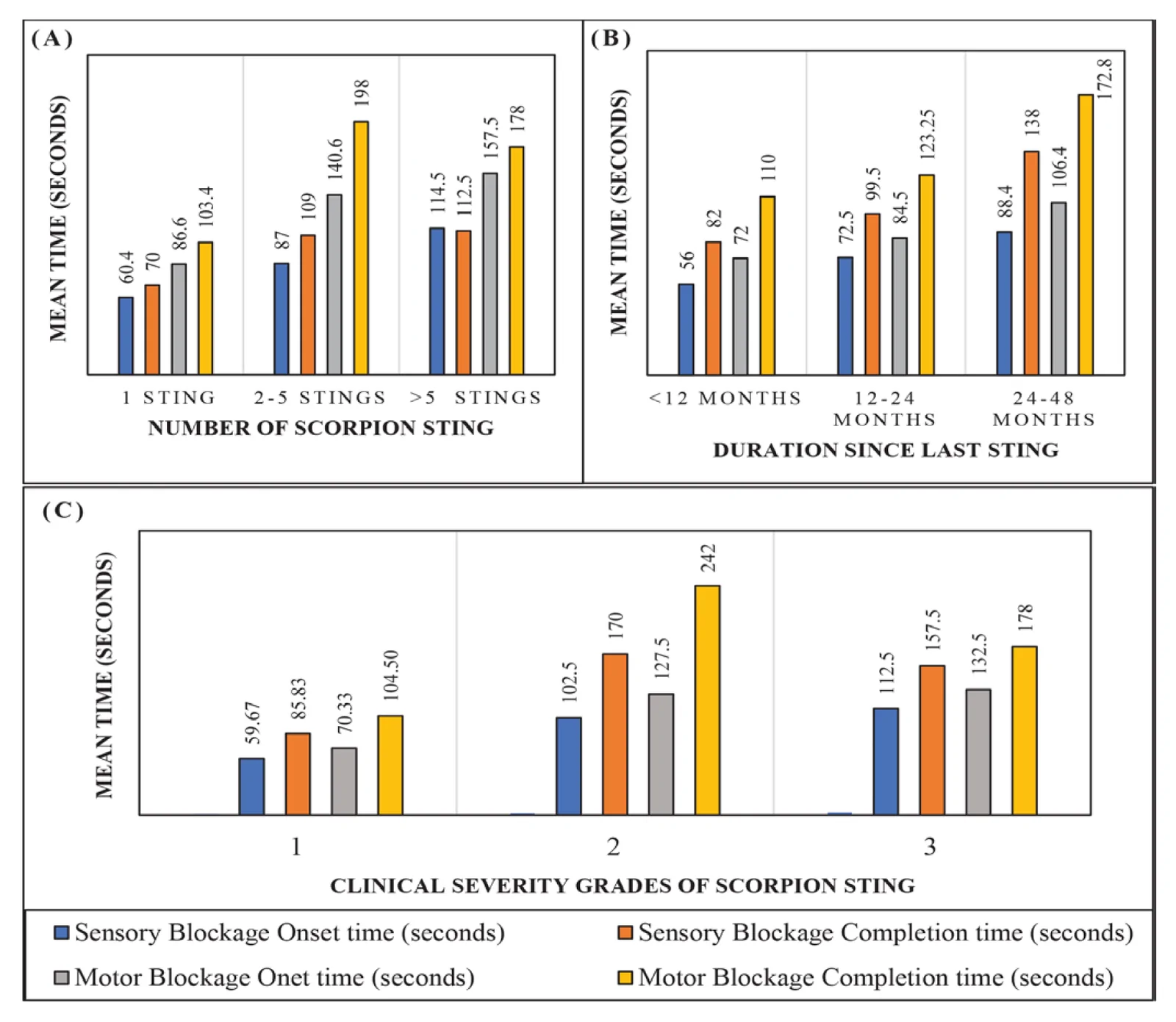
**A:** Association between number of scorpion stings and mean block time. **B:** Association between duration since last scorpion sting and mean block time. **C:** Association between clinical severity grade of scorpion sting and mean block time.

**Figure 2:**
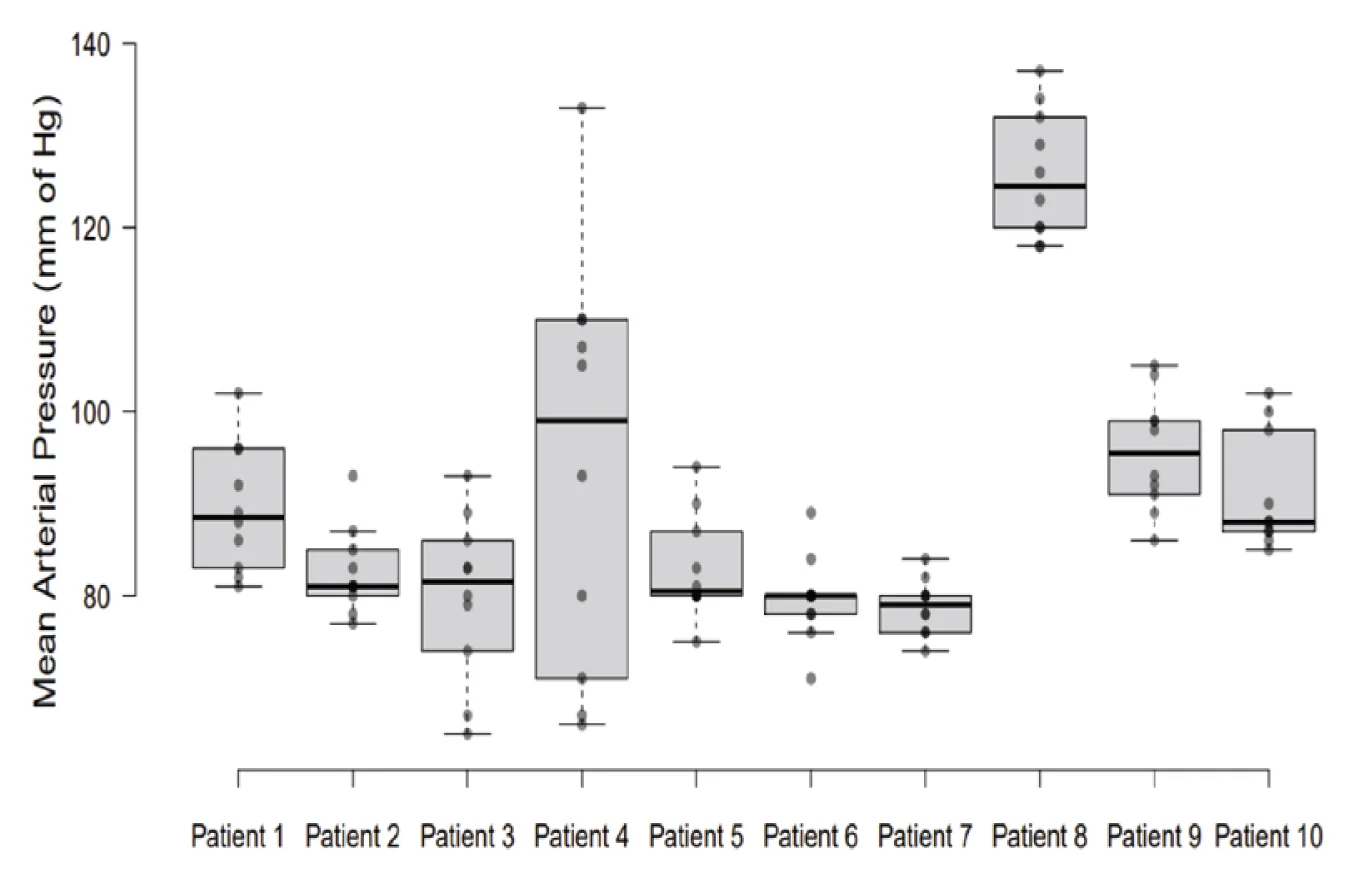
Variation of mean arterial pressure with time in each patient (N = 10).

Out of 10 patients, 5 patients had a history of a single sting, 3 patients had 2–5 stings and 2 patients >5 stings. The mean time to onset and completion of sensory and motor blockage was more in patients with history of multiple (>2) stings [Figure 1A]. Patients with a sting within 1 year had relatively faster onset of sensory and motor block as compared to patients with a sting between 1–5 years [Figure 1B]. Patients with clinical grade 2 and 3 stings had relatively delayed onset and completion of blockage [Figure 1C]. No episode of post-spinal hypotension (fall in mean arterial pressure [MAP] >20% from baseline) was observed in the current patients, except patient 4 [Figure 2].

## Discussion

Scorpion stings are a common occurrence in India. Usually harmless, with manifestations such as severe pain and burning sensation at the site of sting. Systemic manifestations such as myocardial infarction, acute pulmonary oedema, cardiogenic shock and death are very rare.^8^ Thus, a large rural population coming for elective surgical procedures, may give history of grades 1 or 2 stings.^6^

Scorpion venom is a weak acid (pH 6.5) and a highly complex mixture of salts, nucleotides, biogenic amines, enzymes, mucoproteins and neurotoxins, acting on ion channels specifically voltage gated sodium channels (VGSC). Out of various scorpion toxins, alpha and beta toxins are known to bind to mammalian VGSC. The alpha toxin binds extracellularly to S3–S4 loop in domain IV and extracellular part of segment S5–S6 of domain I.^9^ The beta toxin binds to extracellular part of segment 4 of domain II.^9^ The binding site of local anesthetics (LA) is segment 6 of domain IV of the alpha subunit of VGSC.^10^ Panditrao *et al*. had described the resistance to intrathecal bupivacaine in patients with a history of scorpion sting and postulated that scorpion toxin itself or the antibodies against the toxin are responsible for the development of resistance to intrathecal bupivacaine.^2^^,^^3^

Amrita *et al*. demonstrated adequate sensory and motor block after SAB with 0.75% hyperbaric ropivacaine in 2 patients with a history of scorpion sting with documented resistance to bupivacaine on subcutaneous LA testing.^11^ Similarly, the present case series demonstrated successful SAB with 0.75% hyperbaric ropivacaine in 10 patients with a history of scorpion sting. The mean time to onset and completion of sensory and motor blockage was more in patients with history of multiple (>2) stings as compared to a single sting. This may be due to the antibodies against scorpion venom that had accumulated with multiple stings as postulated by Panditrao *et al*.^2^^,^^3^

Patients with sting history between 1–5 years had comparatively delayed onset and completion of sensory and motor blockage as compared to patients with a sting within 1 year. Patients with clinical grade 2 and 3 stings had relatively delayed onset and completion of sensory and motor blockage.

Molecular modelling of local anaesthetic binding with VGSC has demonstrated the differences in the relative alignment of aromatic part of ropivacaine as compared to other LA on VGSC. The aromatic part of ropivacaine aligns towards the outer side of VGSC whereas the aromatic part of bupivacaine aligns towards the inner side of the channel.^12^^,^^13^ This differential alignment of the aromatic ring may contribute to the difference in resistance of the two LA caused by scorpion sting. Furthermore, action of ropivacaine on gamma aminobutyric acid A and N-methyl-D-aspartate receptors, facilitates its LA action, thereby decreasing the chances of its resistance in patients with a scorpion sting.^14^^,^^15^ Thus, differences in the 3D structures of ropivacaine and bupivacaine may confer differences in the activity of their enantiomers in the complex biological environment of the receptors and may be responsible for the success of intra-thecal ropivacaine in patients with scorpion sting.^4^

## Conclusion

Intrathecal hyperbaric ropivacaine may be considered as the local anaesthetic agent of choice in patients with a scorpion sting to prevent failure of sub-arachnoid block. Further scientific studies are needed to further validate these findings.

## Figures and Tables

**Table 1:** Demographic profile and block parameters of patients who presented at a tertiary care hospital of central India with a scorpion sting(s) (N = 10)

Age in years	Gender	ASA-PS	Surgery	Number of times of scorpion sting	Number of stings in past 5 years	Duration since last sting in months	Severity of sting with clinical grade	Sensory blockage onset/ completion in seconds	Motor blockage onset/ completion in seconds
38	Female	1	Fistulectomy	1	1	48	Mild/1	80/135	85/140
62	Male	2	Right inguinal hernia repair	1	1	42	Mild/1	65/90	82/120
38	Male	1	Left femur external fixation	3	2	4	Mild/1	56/82	72/110
70	Female	2	Right proximal femur nailing	1	1	12	Mild/1	39/50	55/67
58	Male	2	Right inguinal hernia repair	6	3	18	Moderate/2	94/140	70/166
26	Male	1	Left inguinal hernia repair	1	1	16	Mild/1	46/68	48/70
56	Male	2	Right inguinal hernia repair	1	1	44	Mild/1	72/90	80/120
50	Male	1	End to end urethroplasty	6	3	48	Severe/3	135/175	155/190
56	Male	2	Right inguinal hernia repair	2	2	22	Moderate/2	115/140	125/190
55	Male	2	Right inguinal hernia repair	4	2	48	Moderate/2	90/200	130/294

ASA-PS = American Society of Anesthesiologists Physical Status.
